# Construction Method of Multimodal 4D Imaging Radar Dataset for Three-Dimensional Traffic Scenes

**DOI:** 10.3390/s26165276

**Published:** 2026-08-20

**Authors:** Zhuanzhuan Zhao, Xin Zhang, Shengyu Yan, Yanze Xue, Yang Liu, Lianqing Zheng, Huiliang Shen

**Affiliations:** 1 School of Automotive Application Engineering, Shaanxi College of Communications Technology, Xi’an 710018, China; 2 School of Automobile, Chang’an University, Xi’an 710018, China; 3 School of Automotive Studies, Tongji University, Shanghai 201804, China; 4 College of Information Science and Electronic Engineering, Zhejiang University, Hangzhou 310027, China

**Keywords:** automotive engineering, 4D imaging radar, three-dimensional traffic scenario, View of Delft (VoD), large model annotation, time synchronization

## Abstract

The latest generation of 4D imaging radar demonstrates significant potential in autonomous driving environmental perception, leveraging its capability to provide target elevation data and dense point clouds. This paper introduces a complete method for constructing a multimodal 4D imaging radar dataset for three-dimensional traffic scenes. It illustrates the hardware and software configurations of the data-acquisition vehicle. Methods including multi-sensor coordination, parameter calibration, timestamp synchronization and spatial datum synchronization are proposed. And eight typical three-dimensional traffic scenarios are designed, such as rainy weather environments, dense heterogeneous targets, enclosed tunnels, high-speed cut-in of multiple vehicles, multi-layered stereoscopic structures and edge working condition reproduction. In addition, this paper puts forward a frame-by-frame processing method for high-resolution images and point cloud data collected by the high-definition camera-LiDAR-4D imaging radar collaborative system. A large model-based 3D annotation method for multiple types of targets is proposed, generating a spatio-temporal sequence-optimized four-dimensional annotation sequence, and finally constructs a complete and high-quality multimodal 4D imaging radar dataset for three-dimensional traffic scenes. The results show that the constructed dataset enables the synchronization of timestamps and spatial coordinate systems. The large model can achieve high-precision 3D annotation for the four predefined target types. The dataset contains 11,400 frames of data from high-definition cameras, LiDAR, and 4D imaging radar, with 131,642 labels. This study will provide reliable fundamental support for the training and verification of 4D imaging radar perception algorithms, vehicle decision-making and planning in complex scenarios, and multi-sensor fusion technologies.

## 1. Introduction

Currently, autonomous driving technology is attracting significant attention and continues to undergo rapid development and iteration [1,2]. Constructing precise and robust environmental perception systems is central to realizing high-level autonomous driving [3]. Mainstream solutions currently rely on multi-sensor fusion technologies such as cameras and LiDAR, i.e., Light Detection and Ranging devices [4,5,6]. However, the perception performance of such solutions can deteriorate significantly under adverse weather conditions like rain, snow, and fog, as well as under sudden illumination changes [7]. Laser beams are scattered or absorbed by suspended particles in the atmosphere, leading to shortened detection ranges, sparse point clouds, or even sensor failure. This has emerged as a significant challenge constraining the safe deployment of autonomous driving functionalities [8].

Millimeter-wave radar (Radar) can compensate for these shortcomings due to its robust environmental adaptability and unique advantages in measuring target velocity [9]. Traditional 3D millimeter-wave radar suffers from low resolution and a lack of height information, making it difficult to effectively classify static obstacles and limiting its performance in complex three-dimensional traffic scenarios. In contrast, the new generation of high-resolution 4D imaging millimeter-wave radar (4D imaging radar) adds an elevation measurement dimension based on azimuth and range measurements. This enables the generation of 3D point clouds containing precise velocity information. Such high-density point clouds significantly mitigate the limitations of traditional radar, demonstrating promising potential for advanced tasks such as target detection and tracking, as well as drivable area segmentation [10]. It is characterized by high robustness and low cost.

To harness the potential of 4D imaging radar and develop robust perception algorithms, large-scale, diverse, and publicly available high-quality datasets with 2D or 3D annotations are indispensable. Both academia and industry have released a series of valuable datasets. For instance, nuScenes [11], as an early large-scale multimodal dataset, contains radar data. However, its radar resolution is relatively low, and it is primarily designed for advanced driver-assistance systems (ADASs). To address the challenge of adverse weather, the RADIATE [12] dataset was introduced, focusing on the collection of high-resolution radar data under conditions such as dense fog, rain, and snow, supporting all-weather perception research. With technological advancements, more datasets are now emphasizing high-resolution radar. For example, RadarScenes [13] provides millimeter-wave radar point clouds with 2D semantic annotations, facilitating fine-grained scene understanding. Datasets such as RADIal [14] provide raw ADC (Analog-to-Digital Converter) data from radar, enabling deep learning research from the signal processing stage onward. The VoD [15] dataset focuses on complex urban traffic scenarios, particularly the detection of vulnerable road users (VRUs). It provides rich 3D bounding box annotations, including numerous instances of pedestrians, cyclists, and other VRU categories. Its data is released as radar point clouds, containing range, azimuth, elevation, and Doppler information. Furthermore, to address China’s complex traffic environment, TJ4DRadSet [16] has released 4D imaging radar point cloud data, along with high-quality 3D annotations, further advancing research on 4D imaging radar perception algorithms. The comprehensive comparison of mainstream radar datasets for autonomous driving is shown in Table 1.

Although existing datasets, including TJ4DRadSet, have advanced research in related fields, they still exhibit certain limitations. Many of these datasets primarily employ traditional low-resolution radar, failing to capture the advantages of 4D imaging radar. Alternatively, they may lack comprehensive scenario coverage, omitting specific regions or extreme operational conditions such as adverse weather, highway environments, pedestrian crossings, and poorly lit tunnels. Furthermore, the scale and continuity of the annotated data may not fully meet the requirements of high-level autonomous driving algorithms, especially those based on deep learning methods.

To mitigate the above gaps and further advance the application and development of 4D imaging radar in autonomous driving perception, this paper presents a comprehensive methodology for constructing a three-dimensional traffic scenario repository. Centered on a high-resolution 4D imaging radar as the primary sensor, and using high-resolution cameras and LiDAR, the aim is to build a multimodal, high-quality dataset tailored for highway and complex urban three-dimensional traffic scenarios.

## 2. Data-Acquisition Scheme

### 2.1. Data Collection Vehicle Configuration

**Figure 1:**
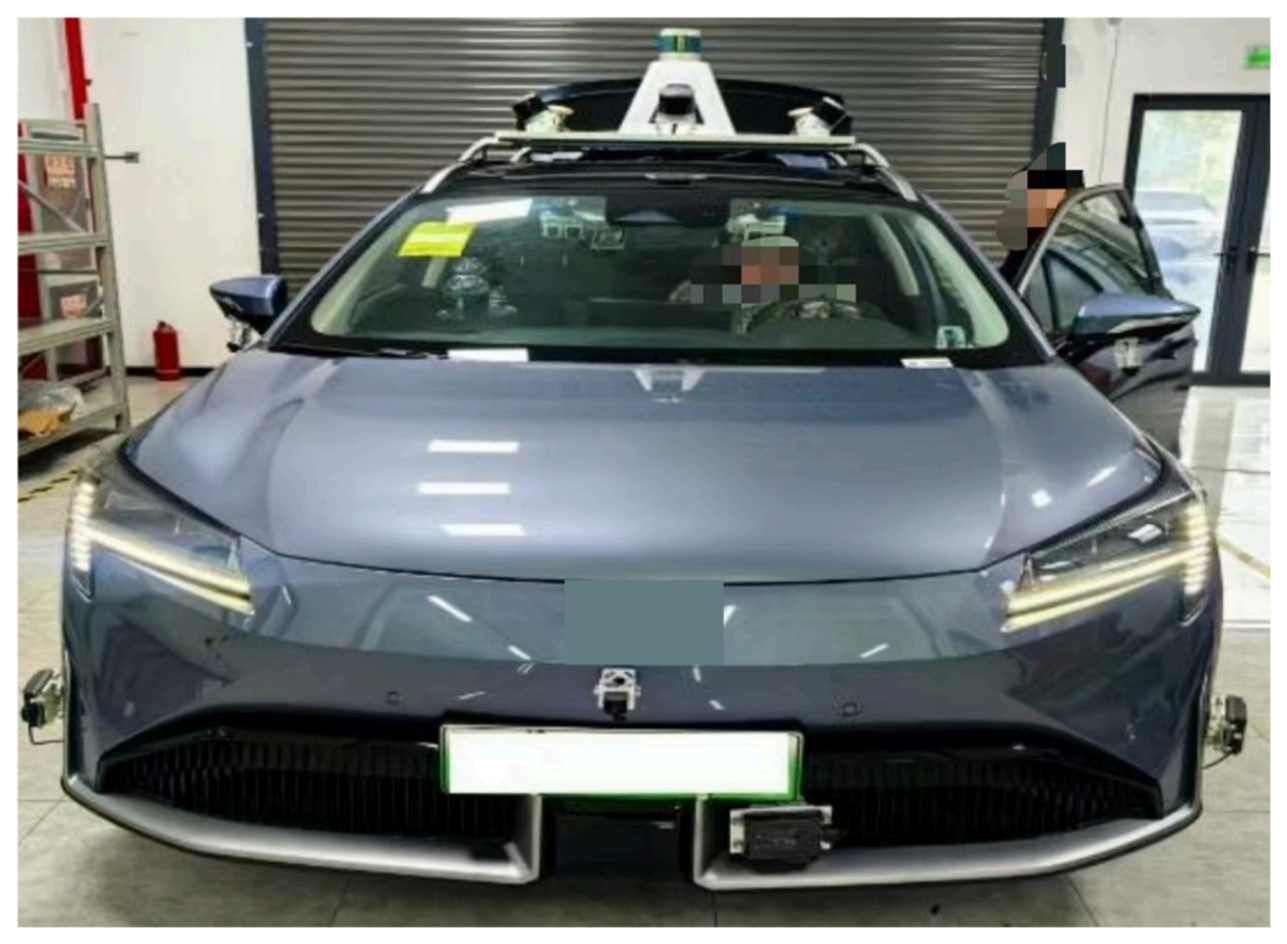
Data collection vehicle for the high-quality dataset.

A modified pure electric vehicle was employed, integrated with a comprehensive suite of high-performance core sensors, including a 4D imaging radar, as shown in Figure 1. This system can synchronously activate all sensor drivers to collect and store, in real time, multimodal raw point cloud data, including frame-by-frame high-resolution images and point clouds. The mounting positions of all sensors were meticulously designed and calibrated to ensure optimal perceptual coverage, minimal field-of-view occlusion, and high-quality data transmission and feedback.

The specific models and parameters of the sensors equipped on the data collection vehicle are as follows:

(1) A Senyun SG-AR0820-GMSL2-H60 series high-definition camera was used, with one unit mounted on the windshield and one each at the front and rear license plate positions. The camera has 8 megapixels, a resolution of 3840 × 2160, and a horizontal field of view (HFOV) of 60°.

(2) A HuaCe CGI430 series combined inertial navigation GNSS system was employed, supporting various communication interfaces such as WiFi, serial port, Ethernet port, and CAN. It includes 8 GB of internal storage for saving operational logs.

(3) A Suteng Juchuang RS-Ruby-128 series LiDAR sensor was installed on the roof of the vehicle, providing a 360° field of view, with 128 channels.

(4) The 4D imaging radar sensor utilized is a prototype from Suzhou Haomibo. This 4D imaging radar is based on the Infineon 28 nm chipset, operates in the 76–81 GHz frequency band, and achieves a detection range of up to 400 m. It supports a field of view of ±60° in azimuth and ±15° in elevation. The radar exhibits high-resolution performance: a range resolution of 0.3 m, a velocity resolution of 0.07 m/s, an azimuth angular resolution of 2.5°, and an elevation angular resolution of 1.8°. It can output 2048 points per frame, operates within a temperature range of −40 °C to 85 °C, and has an ingress protection rating of IP65KW. It is compatible with in-vehicle Ethernet/CAN FD communication and is suitable for advanced driver-assistance systems (ADASs) and autonomous driving systems.

(5) An industrial computer from the BRAV-7521 series was employed, running the Ubuntu operating system. It is equipped with an Intel Core i7-8700 processor, 32 GB of RAM, and an NVIDIA RTX 3090 graphics card with 24 GB of GDDR6 VRAM. For storage, it uses a dual-SSD configuration comprising a 2 TB M.2 SSD and a 2 TB SSD.

**Figure 2:**
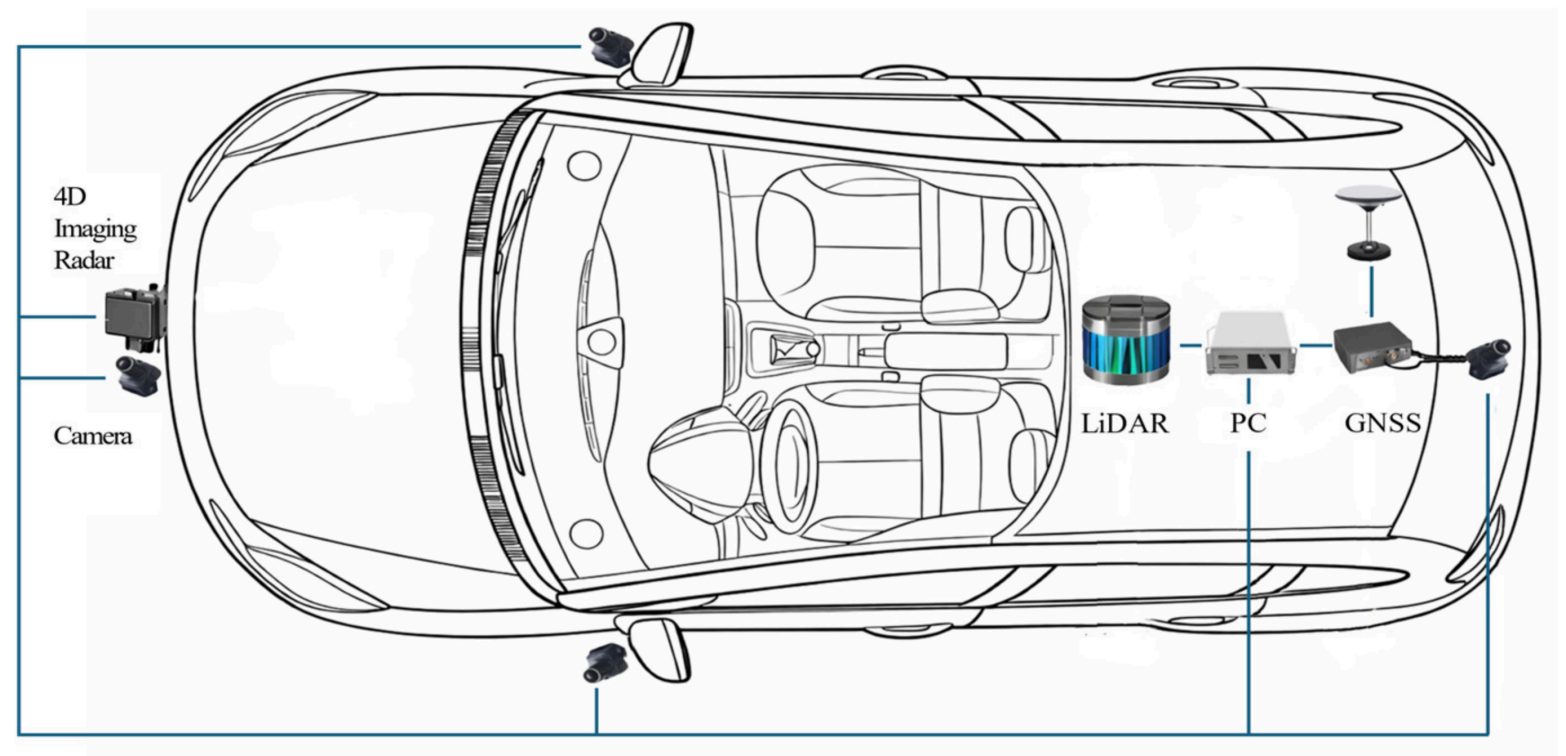
Mounting positions of sensors and the processor.

Mounting positions of sensors and the processor are depicted in Figure 2.

To ensure the accuracy of data acquisition, preparatory work included the following steps:

(1) Hardware and software setup. The hardware equipment comprised a 4D imaging radar, a data-acquisition card, an in-vehicle Ethernet converter, a power supply, etc. The 4D imaging radar was mounted and secured on the data collection vehicle, and its supporting interfaces and communication protocols were inspected. The 4D imaging radar was connected to the data-acquisition card and the in-vehicle Ethernet converter. Software tools for data acquisition, parsing, and Open3D visualization were configured. All connections were verified for correctness, followed by preliminary validation and analysis of the communication. Multiple verification data collections were conducted in different environments to ensure the accurate calibration of the 4D imaging radar and minimize measurement errors.

(2) Data Storage and Security. Safety operating procedures were established to address potential technical failures or other unexpected situations. The collected data were regularly backed up. Encryption measures were implemented during data transmission, and data anonymization was evaluated. The datasets are typically kept confidential or released conditionally under specific agreements after anonymization.

**Figure 3:**
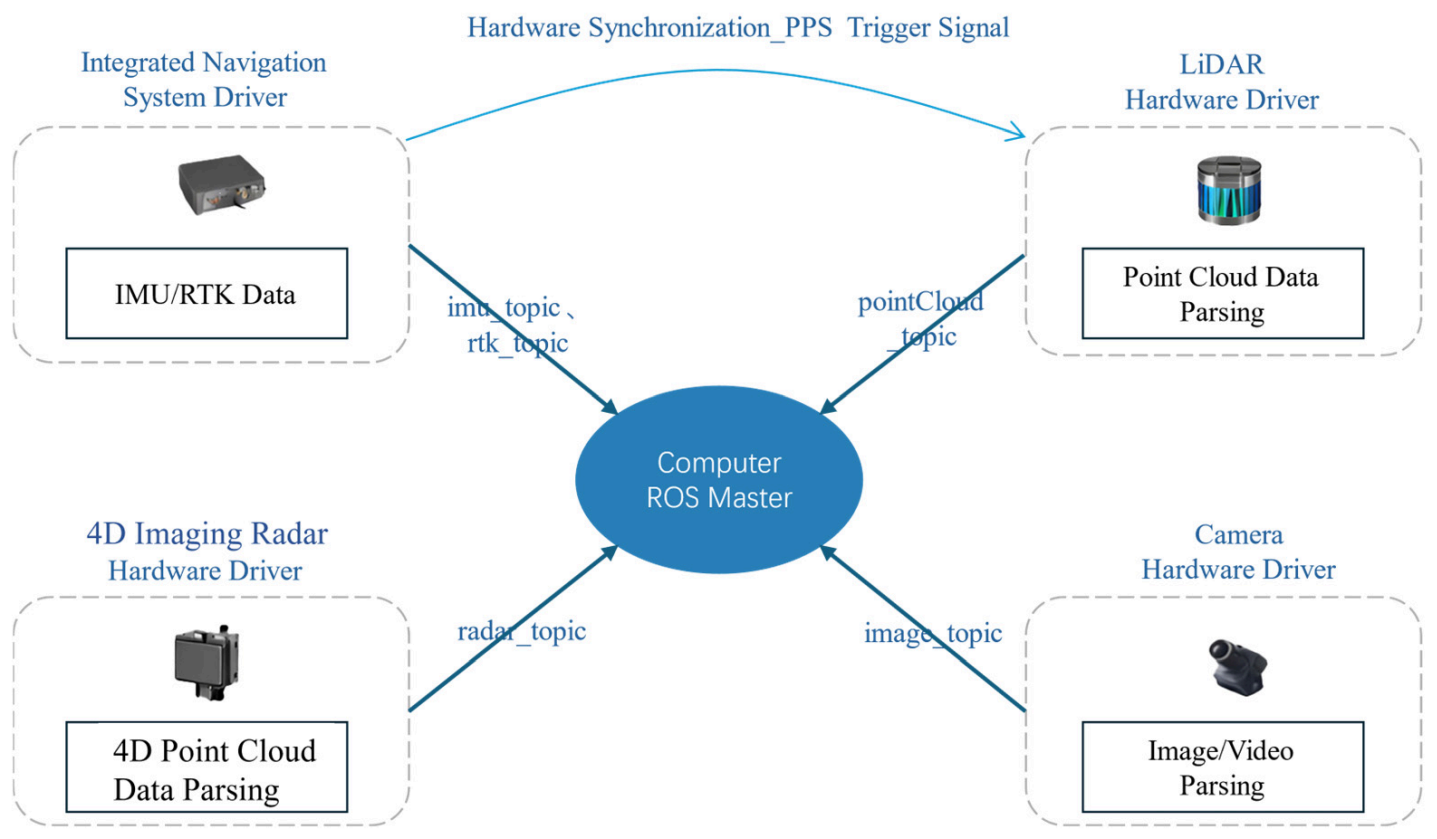
Data transmission link of the sensor system.

The elaborated data transmission link of the sensor system is shown in Figure 3.

### 2.2. Multi-Sensor Synchronization and Calibration

Multi-sensor calibration serves as the foundation for perception algorithms. The sensor calibration process primarily includes intrinsic calibration of the high-resolution cameras, extrinsic calibration between the high-resolution cameras and the LiDAR, and timestamp synchronization. Since the intrinsic parameters of the 4D imaging radar and LiDAR have been factory-calibrated offline, it is only necessary to calibrate the intrinsic parameters of the high-resolution cameras and the extrinsic parameters between the high-resolution cameras and the LiDAR.

(1) System Timing. A high-precision clock synchronization mechanism based on an integrated inertial navigation-GNSS is employed to align the data-acquisition times of all devices. The Global Navigation Satellite System (GNSS) operates at 100 Hz or 300 Hz, enabling the determination of the time offset of each frame relative to the starting point at any given moment. The integrated inertial-navigation–GNSS can receive Global Positioning System (GPS) signals and generate high-precision UTC internally. The GNSS is configured as the master clock, connected to the domain controller (or a clock synchronizer), with one sub-node activated.

(2) Timestamp Synchronization. Other sensors are also connected to the domain controller, with the high-resolution cameras, LiDAR, and 4D imaging radar serving as slave nodes. They are linked to the domain controller via Ethernet, and the Precision Time Protocol (PTP) service is initiated. The PTP protocol automatically synchronizes time between master and slave nodes, enabling the entire data-acquisition system to share a unified, high-precision time reference. This ensures that all data packets generated by the sensors are tagged with hardware timestamps featuring microsecond-level accuracy.

**Figure 4:**
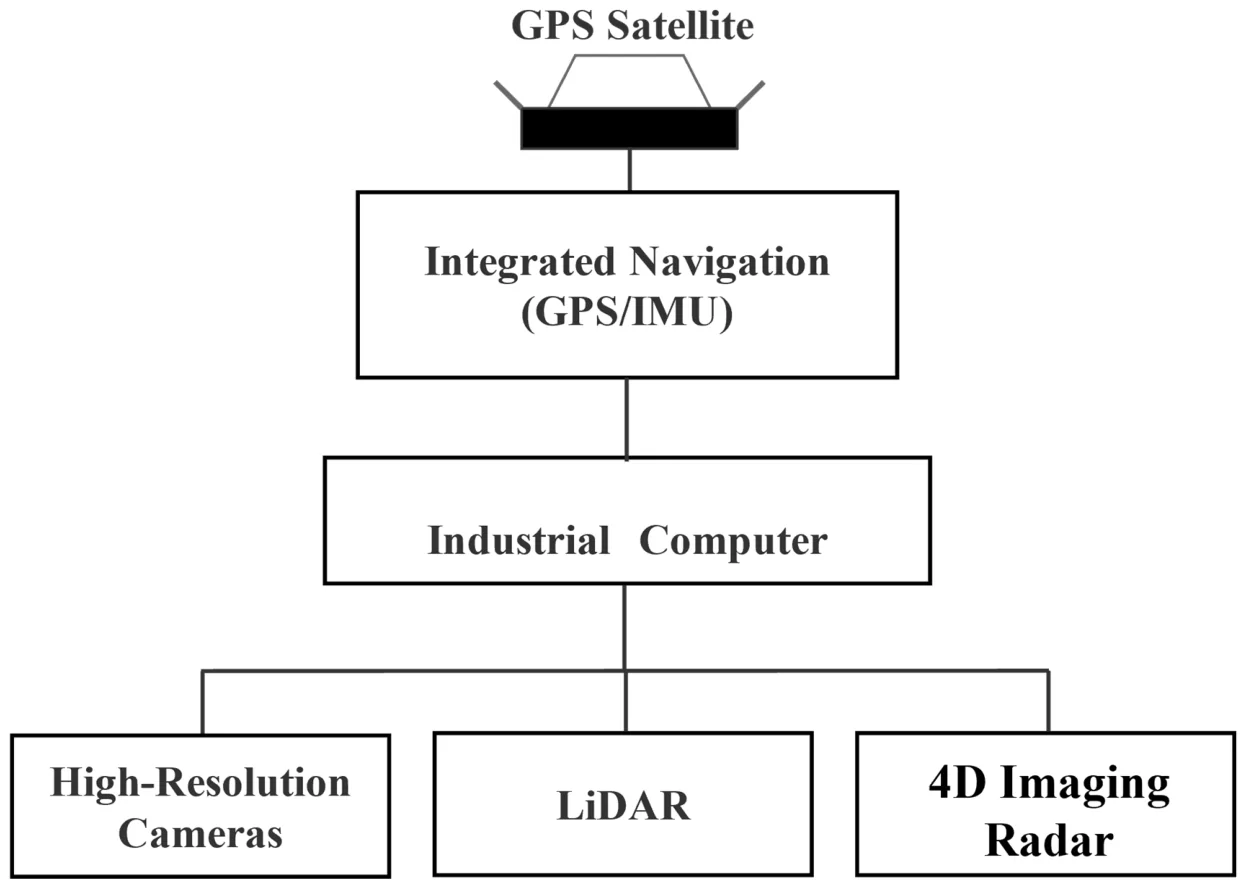
Flowchart of the multi-sensor synchronization scheme.

Figure 4 presents the multi-sensor synchronization scheme flowchart.

Since each sensor operates at a different frame rate—specifically, the LiDAR and high-resolution cameras at 10 Hz and 30 Hz, respectively—the lowest acquisition frequency among all sensors (10 Hz) is adopted as the system’s base acquisition frequency. The unified timestamp is typically anchored to the LiDAR data, given its lowest frequency of 10 frames per second. By setting Δt to 0, the other sensors are synchronized with the LiDAR.

**Figure 5:**
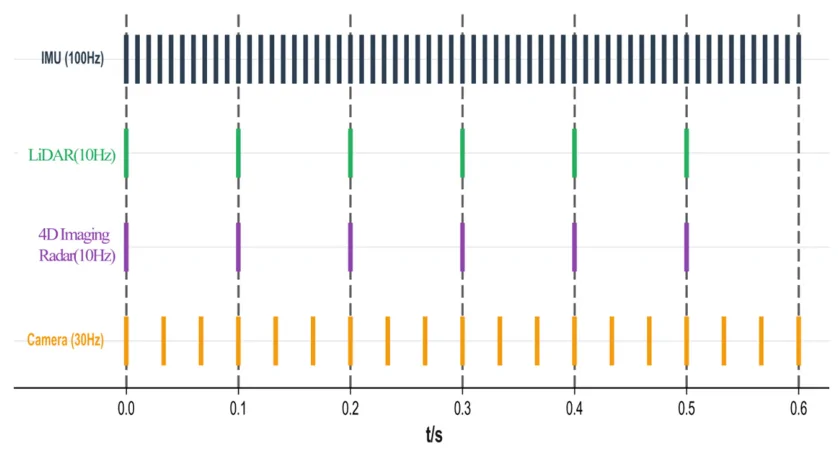
Method for aligning timestamps from different sensors.

Accordingly, a unified timeline is designed to align all data streams using the arrival time of the LiDAR data as the common timestamp reference, as illustrated in Figure 5.

For the high-resolution cameras with an acquisition frequency of 30 Hz, since this is an integer multiple of 10 Hz, frame extraction is performed on the original data at equal intervals. This reduces their data frame rate to 10 Hz while ensuring temporal uniformity. During data post-processing, the timestamp of each LiDAR frame serves as the reference. Corresponding data frames from the other sensors are matched to this timestamp, forming a multimodal data packet precisely aligned in the temporal domain. This process ensures that timestamp errors across all sensors remain at the microsecond level.

When the GPS signal in the tunnel is unstable, the OCXO high-stability crystal oscillator is used to ensure local clock synchronization of each sensor. The IMU and wheel speed odometer are used to determine the spatial coordinates corresponding to each frame of data, and the synchronization accuracy can be controlled within 2 ms in short tunnels [17].

(3) Spatial Reference Synchronization. A crucial step in constructing a comprehensive dataset is the fusion of multimodal data based on precise spatiotemporal calibration results. To achieve accurate fusion of multi-sensor data, rigorous spatial calibration must be completed prior to data collection. This process centers on establishing a unified spatial coordinate reference and accurately computing the rigid-body transformation matrices (rotation matrix R and translation vector T) between the coordinate systems of the various sensors. The vehicle body coordinate system is defined as the global reference frame, with the *X*-axis pointing forward, the *Y*-axis pointing to the left, and the *Z*-axis pointing vertically upward, following the right-hand rule.

During the extrinsic calibration of the high-resolution cameras and the LiDAR, a checkerboard is used to ensure the target is simultaneously detectable by both the camera and the LiDAR. The checkerboard is used to align the point cloud and image data, thereby obtaining the critical extrinsic parameters. The focus should be on calibrating the transformation matrix between the high-resolution cameras and the LiDAR, as well as the relative position and orientation between the 4D imaging radar and the LiDAR. The former is fundamental for accurately projecting 3D point clouds onto 2D images. At the same time, the latter is key to fusing and aligning point cloud data from these two different sources within a common spatial reference frame. The extrinsic parameters between the high-resolution cameras and the LiDAR are obtained via matrix calculations, followed by manual fine-tuning using static objects in the environment.

Once the extrinsic spatial relationships between the sensors are determined, it is also necessary to calibrate the intrinsic parameters of the high-resolution cameras to describe their geometric imaging properties accurately. This involves solving for the intrinsic matrix K, composed of focal lengths and the principal point, as well as the distortion coefficients used to correct lens imaging distortion. Precise intrinsic parameters are fundamental for achieving accurate mapping from the three-dimensional camera coordinate system to the two-dimensional image pixel coordinate system. Based on the precisely calibrated intrinsic and extrinsic parameters, radar point clouds can be projected onto the image plane, achieving pixel-level alignment. This provides a foundation for ensuring geometric consistency in subsequent data fusion and 3D annotation tasks.

For the acquired vehicle POS data, the position of the first frame is determined. The position of the next frame is then calculated by applying an RT4 × 4 transformation matrix relative to the first frame. Since the integrated inertial navigation GNSS typically operates at high frequencies of 100 Hz or 300 Hz, providing high-frequency position updates, the absolute displacement of each frame relative to the starting point can be precisely determined at every moment. Based on the transformation relationship describing the vehicle’s movement from one frame to the next, the points are projected to their corresponding positions using the extrinsic parameters. Ultimately, the point cloud data from both the 4D imaging radar and the LiDAR are projected onto the images.

## 3. Scenario Library Design Oriented Towards 4D Imaging Radar Performance

### 3.1. Core Performance Metrics of 4D Imaging Radar

The core performance metrics of 4D imaging radar during point cloud data acquisition include (see Table 2) velocity, elevation angle, field of view, detection range, etc.

A scenario library should be constructed that covers various conditions, including rainy weather, high-speed driving, enclosed tunnels, high-speed traffic weaving, overpasses, traffic congestion, and complex intersections. In this library, large models are applied for 2D/3D annotation of point cloud data, encompassing four target types: pedestrians, electric bicycles, passenger vehicles, and trucks.

### 3.2. Scenario Library Design

The data collection for the three-dimensional traffic scenario repository presented in this paper was conducted from June to July 2025 in Shanghai, China. The dataset comprises eight representative three-dimensional traffic scenarios (see Table 3), including five urban complex scenarios to test the 4D imaging radar’s data-acquisition performance in three-dimensional environments and three scenarios to evaluate its performance during high-speed driving. Graded recording durations were designed based on the complexity and event density of different scenarios: four standard urban complex scenarios were each recorded for 2 min; one simulated maneuver scenario was recorded for 5 min; and three highway scenarios were each recorded for 2 min.

## 4. The 4D Imaging Radar Point Cloud Data Processing Methodology

### 4.1. Data Processing Pipeline

The acquired 4D imaging radar point cloud data needs to be processed and analyzed to extract valuable information. Each of the eight complex three-dimensional traffic scenarios involves four target categories: pedestrians, electric bicycles, passenger vehicles, and trucks. Data from all sensors are comprehensively recorded in ROSbag files.

Since LiDAR offers higher point cloud density compared to 4D imaging radar, providing more detailed descriptions of 3D object outlines, the annotation system primarily relies on LiDAR point cloud data in conjunction with annotated point clouds and images. Some objects that are occluded or have very sparse LiDAR point returns may still appear in the 4D imaging radar’s field of view due to multipath effects. These can be labeled, with annotations completed either manually or by large models, followed by multiple rounds of review to ensure the dataset’s quality.

(1) Data Preprocessing. The acquired raw point cloud is preprocessed using methods such as the Kalman filter. By utilizing the signal-to-noise ratio (SNR) characteristics output by 4D imaging radar, a dynamic filtering threshold based on 6 dB is set to filter out clutter interference caused by rainy weather environments. Using the 4D imaging radar sDoppler radial velocity and ground height models, elevation outliers and invalid static clutter are eliminated, and false targets generated by tunnel wall reflections are removed.

(2) Point cloud feature enhancement. To address the issue of sparse point clouds in 4D imaging radar, a point cloud feature enhancement method is adopted. On the basis of the anchor point local point cloud completion framework, the neighborhood construction method is improved, and a distance-aware dynamic neighborhood strategy is proposed to replace the fixed K scheme. The algorithm takes the original 4D imaging radar point cloud as input and selects anchor points through farthest-point sampling equalization. The neighborhood search radius is dynamically adjusted based on the anchor detection distance, and a minimum point constraint is set to ensure sufficient geometric information in the neighborhood, thereby enhancing the local expression ability of 4D imaging radar point clouds [18]. The adaptive neighborhood construction algorithm is as follows:

A=FPS(P,M)

$$\forall a_{i}\in A:$$

$$r_{i}={\left\|a_{i}\right\|}_{2},R\left(r_{i}\right)={min(R}_{0}+\alpha \cdot r_{i},R_{max})$$

$$\tilde{N}\left(a_{i}\right)=\left\{\left.p\in P\right|{\left\|{p-a}_{i}\right\|}_{2}<R\left(r_{i}\right)\right\}$$

$$N\left(a_{i}\right)=\left\{\begin{array}{c} \tilde{N}\left(a_{i}\right),\;\left|\tilde{N}\left(a_{i}\right)\right|\;\geq K_{min}\\ \mathrm{TopKNN}\left(P,a_{i},K_{min}\right),\left|\tilde{N}\left(a_{i}\right)\right|<K_{min} \end{array}\right.$$

where A represents the set of anchor points sampled from the farthest points; P represents the 4D imaging radar raw point cloud; M represents the total number of anchor points; $a_{i}$ represents the i-th anchor point obtained from FPS sampling; $r_{i}={\left\|a_{i}\right\|}_{2}$ represents the spatial distance from the anchor point to the radar origin; $R_{0}$ represents basic search radius; α represents distance growth coefficient; $R\left(r_{i}\right)=R_{0}+\alpha \cdot r_{i}$ represents distance-adaptive search radius; $K_{min}$ represents the minimum number of points in the neighborhood; $\tilde{N}\left(a_{i}\right)$ represents the preliminary neighborhood set obtained through radius filtering; and $N\left(a_{i}\right)$ represents the final neighborhood set.

The mainstream evaluation indicators in the field of object detection, Precision and Recall, are used to quantitatively evaluate the improvement effect of point cloud completion algorithms on subsequent object detection tasks.

$$Precision=\frac{TP}{TP+FP}$$

$$Recall=\frac{TP}{TP+FN}$$

where *TP* represents the number of correctly detected targets, *FP* represents the number of incorrectly detected targets, and *FN* represents the number of missed targets.

(3) Target Detection and Tracking. Detection and classification are performed on the 4D imaging radar point cloud data, and target tracking is achieved by leveraging velocity and angle information.

Firstly, the Doppler velocity information of 4D imaging radar is used to distinguish dynamic points from static points, and all selected dynamic points are clustered to obtain initial detection candidate targets. Next, a point-by-point motion estimation module is used to complete the motion information of sparse point clouds, and the estimated point cloud scene flow is used to assist in completing point cloud clusters of the same target between different frames. Finally, the extended Kalman filter is used to estimate the target state, including three-dimensional position, velocity, and acceleration, while performing kinematic consistency checks on the associated trajectories to eliminate false trajectories [19].

**Figure 6:**
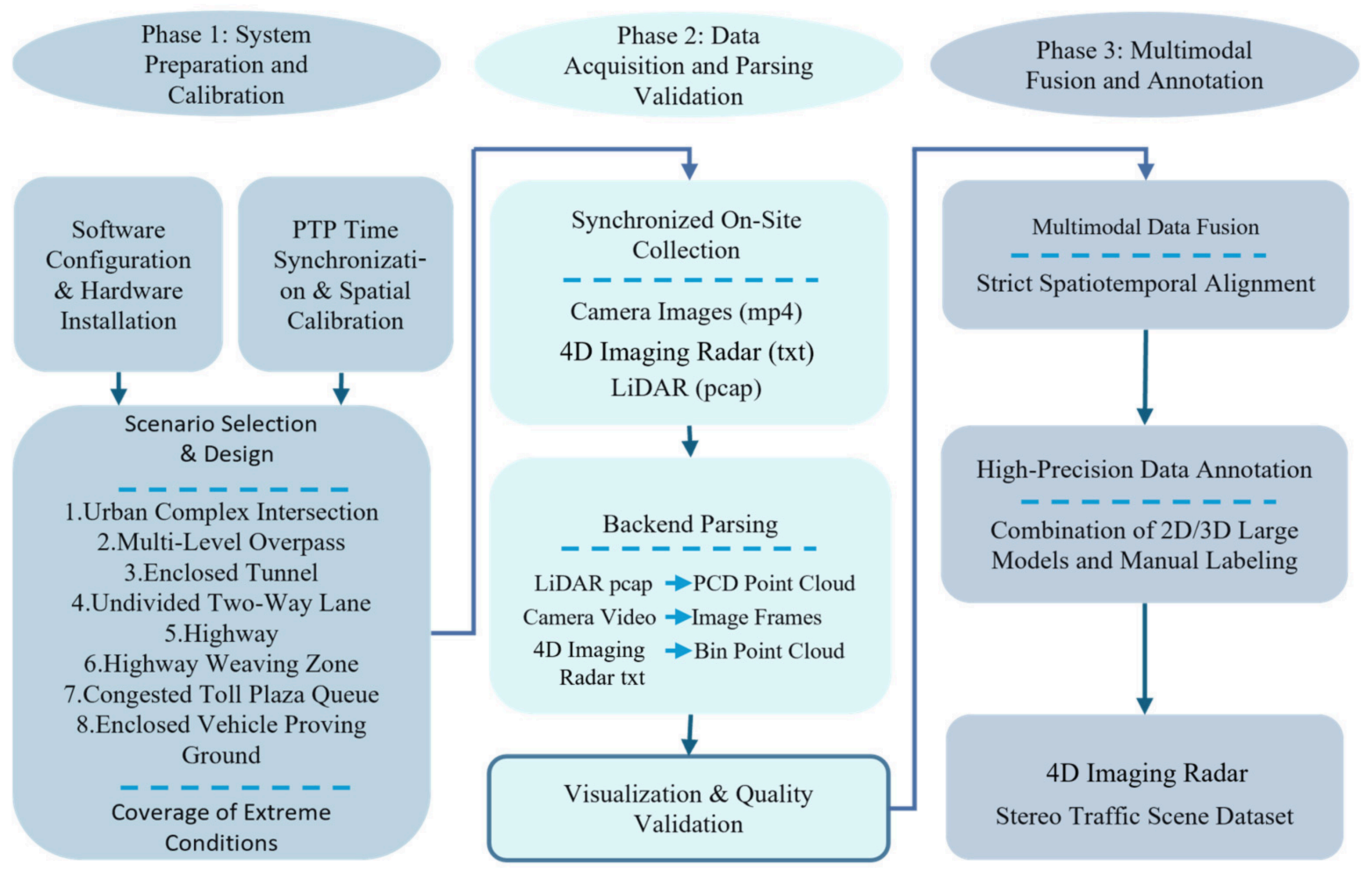
Data processing pipeline for 4D imaging radar point clouds.

The data processing pipeline for 4D imaging radar point clouds is detailed in Figure 6.

The processed data is presented using LiDAR as the temporal reference axis. The data is segmented into 20 s slices, defined by start and end timestamps. For each slice, synchronized data from all other sensors within the corresponding time window is extracted and assembled into a single data package. Following a rule of 0.5 to 1.0 min per package, each package contains approximately 300 to 600 frames.

### 4.2. LiDAR Point Cloud Data Conversion and Verification

**Figure 7:**
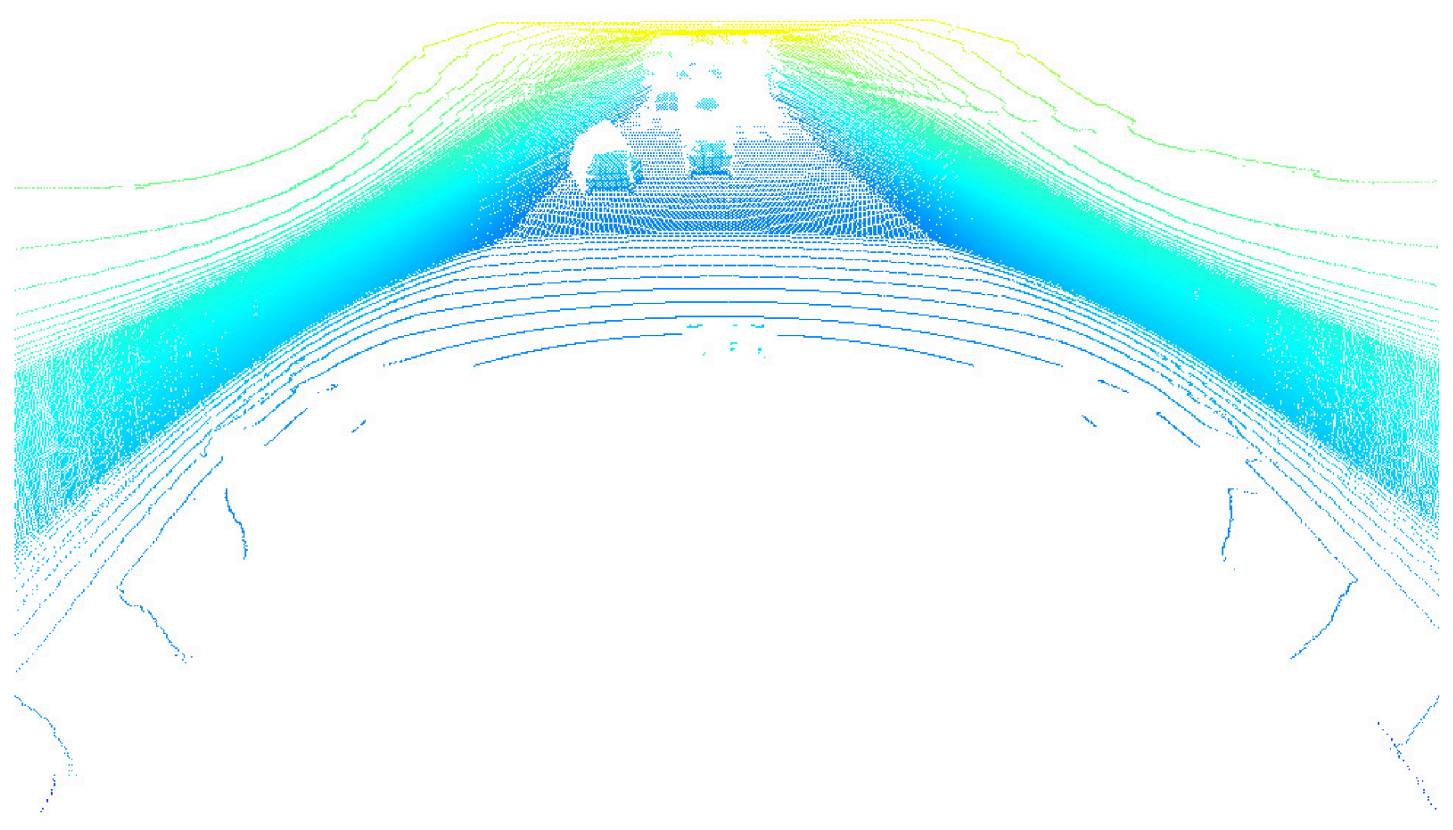
Example of LiDAR PCD point cloud data.

Raw PCAP data were standardized to PCD format for compatibility with mainstream PCL libraries. We developed an Open3D-based visualizer to verify data integrity, supporting per-frame rendering and viewpoint locking to identify geometric distortions or frame loss. Synchronized rendering settings ensure a consistent visual reference across sequences, providing a reliable data foundation for subsequent algorithm evaluation. Sample point clouds are shown in Figure 7.

### 4.3. Processing of 4D Imaging Radar Point Cloud Data

The data post-processing stage aims to parse, format, convert, validate the quality of, and perform multimodal fusion on the massive raw data, ultimately generating a standardized dataset ready for algorithm development. For the 4D imaging radar data, the raw text data packets are first parsed into multiple independent binary BIN files, with each file representing a single frame of point cloud data. Subsequently, specialized scripts parse these BIN files, extracting ten-dimensional information for each point, including Cartesian coordinates (X, Y, Z), raw range, Doppler velocity, azimuth angle, elevation angle, echo power, signal-to-noise ratio (SNR), and ambiguous velocity. To validate data quality, the point cloud data is rendered frame-by-frame in real time into a video from a bird’s-eye perspective, facilitating rapid inspection of target distribution, density, and the plausibility of motion trajectories.

The accuracy of extrinsic parameter calibration can be intuitively verified by observing the alignment between the point cloud data and the object contours in the images. This also supports subsequent visual and point cloud fusion algorithms, such as 3D object detection. By fusing LiDAR and 4D imaging radar point cloud data, a unified three-dimensional environmental model is constructed, containing precise positions, dense contours, and reliable velocity information. The core of this process involves applying the LiDAR-4D imaging radar extrinsic transformation matrix to convert the 4D imaging radar point cloud data into the LiDAR coordinate system. This achieves precise alignment of the two point cloud datasets under a common spatiotemporal reference, generating a multimodal sensor dataset with timestamps strictly synchronized and multi-sensor data aligned within a hierarchically unified coordinate system. The fused dataset synergistically leverages LiDAR’s high-precision spatial modeling and 4D imaging radar’s velocity perception, providing complementary information for target tracking under adverse weather conditions.

**Figure 8:**
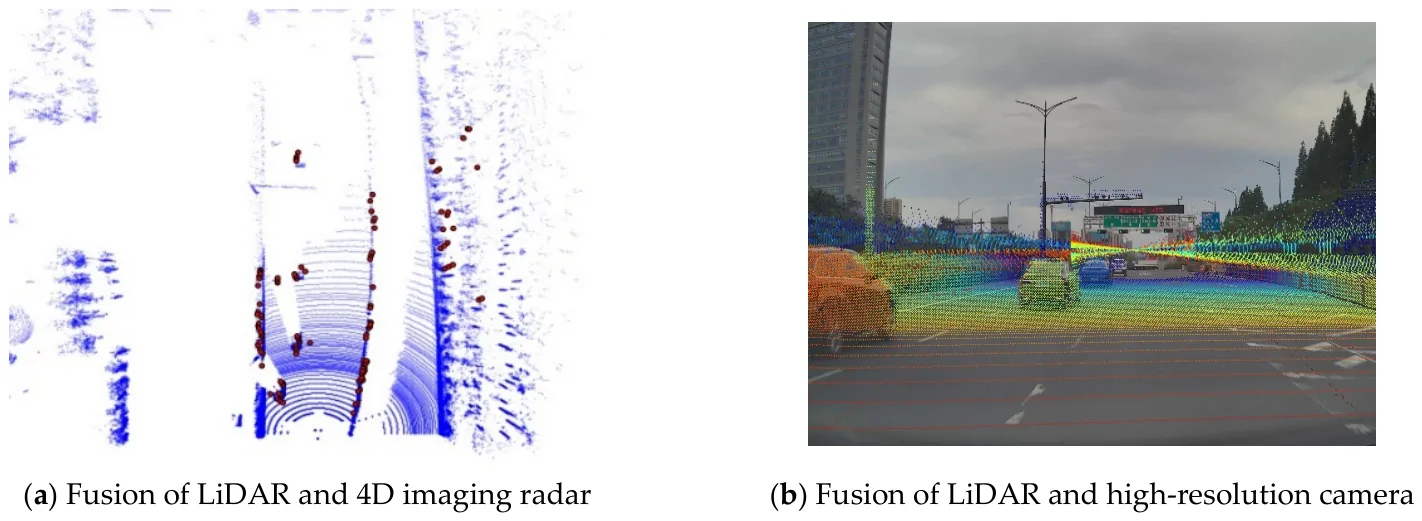
Multi-sensor data fusion and visualization.

Multi-sensor data fusion and visualization of LiDAR point cloud and 4D imaging radar is illustrated in Figure 8a, while that of LiDAR and high-resolution camera is shown in Figure 8b.

### 4.4. Quantitative Assessment of Data Fidelity and Sensing Potential

We performed a statistical analysis across the full 11,400-frame sequence. Doppler-spatial consistency was applied to filter out environmental clutter and static reflections. The analysis yields an average moving-point ratio of 40.06% and a mean detection count of 3.45 dynamic objects per frame (peaking at 15). These results suggest that the preprocessing preserves motion information in complex traffic scenes while mitigating static interference.

## 5. The 4D Imaging Radar Point Cloud Data Annotation via Pre-Trained 3D Detection Model

### 5.1. Automated Annotation and Trajectory Optimization

**Figure 9:**
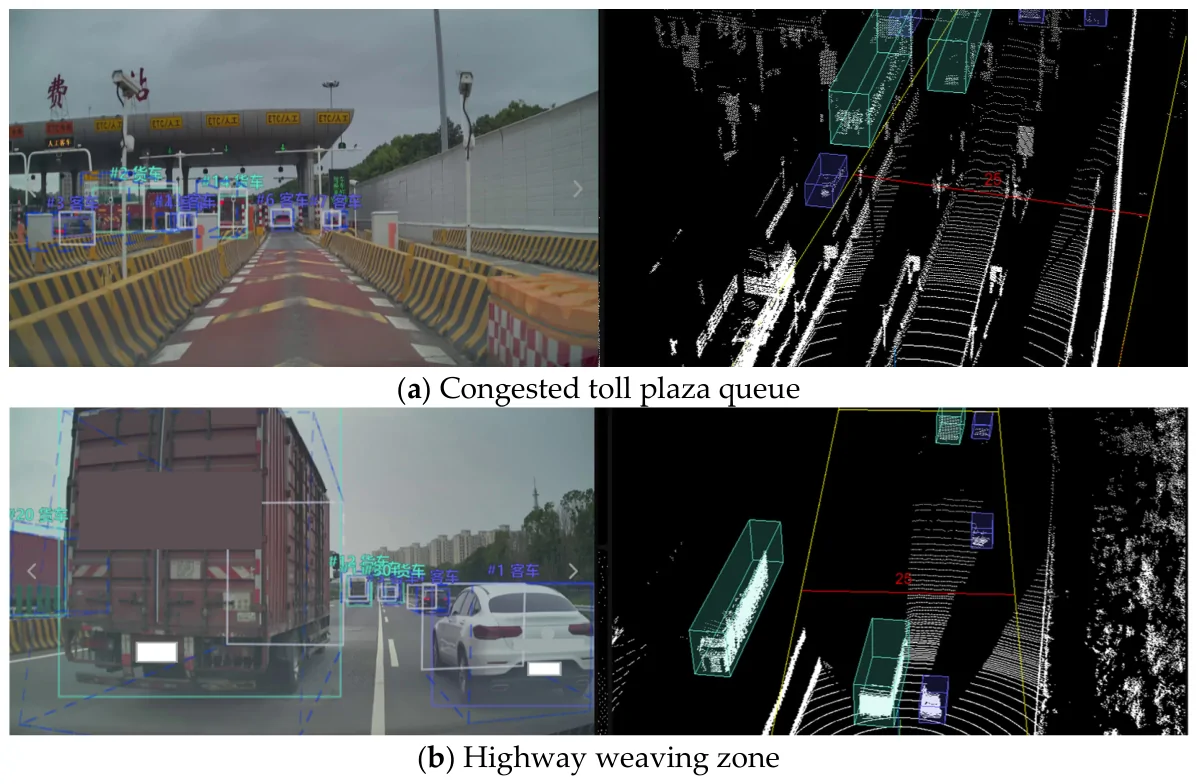

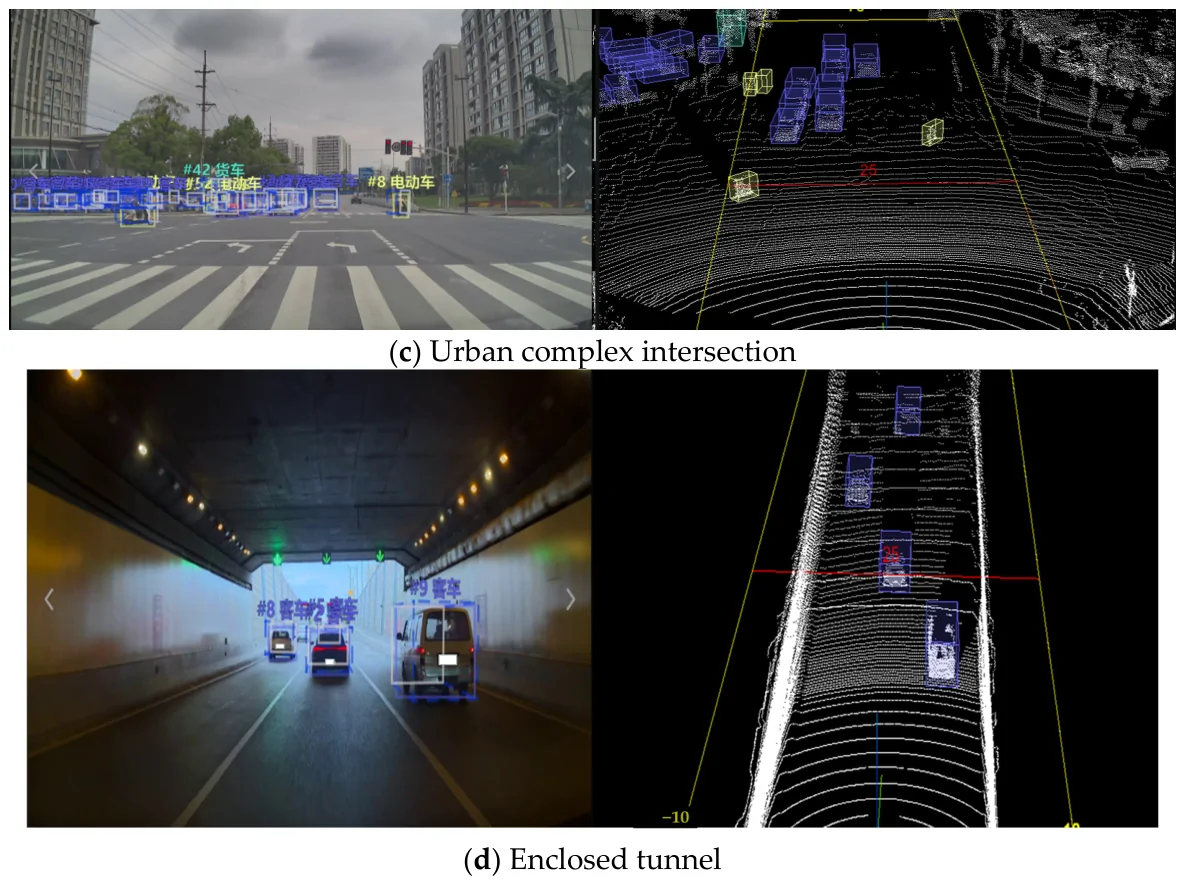
The annotation results of the pre-trained 3D detection model for selected scenarios.

The real-world 3D annotations in the three-dimensional traffic scenario repository primarily include bounding boxes, categories, and trajectory IDs for each object. To construct a dataset capable of precisely evaluating multimodal perception algorithms, automated annotation is performed by leveraging a powerful, pre-trained 3D detection model. Figure 9 shows the annotation results of the pre-trained 3D detection model for selected scenarios.

In Figure 9, the blue box represents passenger vehicles, the green box represents trucks, and the yellow box represents electric bicycles.

#### 5.1.1. Pre-Trained 3D Detection Model

The pre-trained 3D detection model is an offline 3D automatic annotation model formed by pre-training a large amount of LiDAR point cloud data based on CenterPoint. The annotation is mainly based on the generation of LiDAR point clouds and projected onto the image and the LiDAR coordinate system through extrinsic projection. This model is not a large language model (LLM), so there will be no prompts.

The detailed rules for point cloud annotation are as follows:

(1) Annotate point cloud data based on reference images, divided into six perspectives including front, back, left, right, etc.

(2) Tracking annotation: the same vehicle in the same entry needs to maintain a consistent ID.

(3) Labeling closely follows the contour of the obstacle, and if the obstruction exceeds the type that cannot be recognized by the naked eye, no labeling is required.

(4) Label the vehicle with a 3D frame and indicate the orientation of the front and head of the vehicle.

(5) If the number of target points is less than 10, do not label them.

#### 5.1.2. Three-Dimensional Annotation Algorithm and Process

(1) 3D Annotation Algorithm

We adopt a 3D object annotation method based on adaptive iterative control. Based on the semi-automatic annotation framework of keyframe interpolation, the effectiveness of annotation is jointly verified by point cloud registration confidence and target motion constraints. At the same time, an adaptive iteration termination strategy is introduced to identify and optimize stagnant states based on changes in confidence, achieving early exit. Combined with a maximum iteration limit and an optimal box fallback fault-tolerant mechanism, it effectively improves the problems of boundary box mismatch and temporal jitter caused by traditional single interpolation optimization while balancing annotation accuracy and computational efficiency [20]. The algorithm is as follows:

$$Z_{final}=F\left(S,M_{pre},M_{refine},\xi_{conf},P\right)$$

K=SelectKeyFrames(S)

$$B_{init}=M_{pre}\left(K\right)$$

$$B_{mr}=ManualRefineBBoxwithID\left(K,B_{init}\right)$$

$$B_{tmp}=Interpolate\left(B_{mr},P,f_{k}\right)$$

$$B_{refine}=M_{refine}\left({B_{tmp},f}_{k}\right)$$

$$\left\{\begin{array}{c} {Condition}_{1}:conf\geq \xi_{conf}⋀a_{cal}\leq a_{thresh}⋀\omega_{cal}\leq \omega_{thresh}\\ {Condition}_{2}:\Delta conf<\varepsilon \cup ⋁stagnate\text{\_}cnt\geq R\\ {Condition}_{3}:iteration\geq {Iteration}_{max} \end{array}\right.$$

where $Z_{final}$ represents the final annotation result; S represents keyframe sequence; $M_{pre}$ represents the pre-annotated model; $M_{refine}$ represents the refinement model; $\xi_{conf}$ represents confidence threshold; P represents pose information; $B_{init}$ represents initial bounding boxes of keyframes; $B_{mr}$ represents manually calibrated keyframe reference bounding boxes; $B_{tmp}$ represents interpolated initial bounding boxes; $B_{refine}$ represents iteratively refined bounding boxes; conf represents confidence level; max_conf represents maximum confidence level during iterations; Δconf represents confidence increment between adjacent iteration rounds; ε represents effective increment threshold of confidence; R represents the allowed number of continuous stagnation rounds; iteration represents current iteration count; ${Iteration}_{max}$ represents maximum iteration limit; $a_{cal}$, $\omega_{cal}$ are the calculated acceleration and angular velocity, respectively; $a_{thresh}$, $\omega_{thresh}$ are the thresholds of acceleration and angular velocity, respectively; best_bbox is the optimal bounding box during iterations.

In this article, ε is set to 0.01, R is set to 2, and ${Iteration}_{max}$ is set to 5.

(2) 3D Annotation Process

The model’s workflow for generating 3D object annotations is as follows:

(a) Select a uniformly spaced subset of keyframes from the input LiDAR sequence and generate initial bounding boxes using the pre-trained 3D detection model.

(b) Manually refine these initial boxes and assign them unique identifiers. Specifically, manually adjust the target category, 3D center, size, yaw angle, and trajectory ID to form high-precision keyframe annotations.

(c) After establishing the keyframe skeleton, the process proceeds to automated post-processing and quality optimization, the core of which lies in temporal interpolation and iterative refinement.

(d) For the vast number of non-keyframes between keyframes, the system first utilizes ego-vehicle pose information from high-precision GNSS to generate initial bounding box predictions through a temporal interpolation algorithm.

(e) A refinement network based on an encoder–decoder architecture then performs iterative optimization on these interpolated results.

During each iteration, the system evaluates the optimization quality using two core metrics: first, the registration confidence between the point cloud within the current bounding box and the target point cloud from the reference keyframe, calculated using the Iterative Closest Point algorithm; second, a consistency check on the target’s kinematic parameters performed in conjunction with a Kalman Filter. This kinematics-based plausibility verification mechanism effectively filters out motion trajectories that violate physical laws, ensuring the smoothness and authenticity of the annotated trajectories across spatial and temporal dimensions. Ultimately, it outputs a four-dimensional annotation sequence optimized for spatiotemporal consistency.

### 5.2. Manual Verification

#### 5.2.1. Manual Verification Content

Although the annotation pipeline through the pre-trained 3D detection model is highly efficient, to ensure the accuracy of ground truth data in complex scenarios (such as adverse weather, severe occlusion, or dense traffic flow), all algorithm-processed data must undergo a final, human-in-the-loop review. Specialized annotators utilize a customized 4D (spatiotemporal) annotation platform to perform a 100% visual inspection of all model outputs. This large-scale platform was built on a powerful interactive environment that supports the visualization of multi-view sensor data, including the synchronized projection and playback of LiDAR point clouds and high-definition camera videos, enabling the visualization and editing of complete trajectories for all four target types.

To enhance the accuracy and efficiency of the review process, the large-model platform used 3D bounding boxes in distinct colors and sizes to visually differentiate among target categories. For instance, pedestrians are represented by blue boxes, passenger vehicles by yellow boxes, and so forth. This enables reviewers to identify potential classification errors quickly. They conduct detailed inspections frame by frame and target by target, focusing on correcting sporadic errors that the automated pipeline struggles to avoid, such as logical issues like ID switches, misclassifications, and trajectory interruptions. Furthermore, they perform fine-grained sub-voxel adjustments to 3D bounding boxes, ensuring that the boxes precisely encompass the target point cloud contours in every frame and thereby achieving high geometric accuracy.

#### 5.2.2. Annotation Quality Control

To ensure the quality of annotation, the following quality control mechanisms are established:

(1) Full sequence review: Manual correction mainly synchronizes the viewing of LiDAR point clouds, images, and complete target trajectories. The review focuses on issues such as missed markings, 3D frame position and size errors, yaw angle errors, ID switching, etc. Due to the fact that each sequence undergoes frame-by-frame review, the annotation is considered by humans to achieve satisfactory accuracy.

(2) Consistency verification: Randomly select some keyframes and have them relabeled by two independent annotators. For the annotation results *B1* and *B2* of the same target, the Intersection over Union (*IoU*) is used as the consistency metric:

$$IoU=\frac{\left|B_{1}⋂B_{2}\right|}{\left|B_{1}⋃B_{2}\right|}$$

When *IoU* > 0.7, the annotation is defined as consistent.

(3) Annotation efficiency index: The Average Center Translation Error (*ACE*) and Average Rotation Error (*ARE*) are adopted to quantify the annotators’ correction workload:

$$ACE=\frac{1}{N}\sum_{i=1}^{N}{\left\|c_{i}^{pred}-\left.c_{i}^{gt}\right\|\right.}_{2}$$

where $c_{i}^{pred}$ and $c_{i}^{gt}$ are the predicted center and ground-truth center of the i−th target, respectively, and ${\left\|\left.\cdot \right\|\right.}_{2}$ denotes the Euclidean distance.

$$ARE=\frac{1}{N}\sum_{i=1}^{N}{\left\|\theta_{i}^{pred}-\left.\theta_{i}^{gt}\right\|\right.}_{2}$$

where $\theta_{i}^{pred}$ and $\theta_{i}^{gt}$ are the predicted yaw angle and ground-truth yaw angle, respectively.

*ACE* and *ARE* directly reflect the degree of deviation between the predicted results and the ground truth values; The smaller the deviation, the less manual calibration work is required.

Through the comprehensive pipeline described above—from large-model 3D annotation to refined manual verification—precise and refined annotation of 11,400 key frames was completed, generating 131,642 four-dimensional dynamic target labels with high-precision temporal information. These annotation results provide a sufficiently large and highly reliable validation and training data base for the subsequent performance evaluation and technical iteration of 4D imaging radar perception algorithms.

### 5.3. Baseline Experiments

To test the dataset’s capability to support the 3D object detection task of 4D imaging radar, PointPillars was used as the baseline for 4D imaging radar detection. This experiment selects cars and trucks with relatively sufficient radar echoes as the detection categories. The experimental data is divided into a training set and a testing set. The testing set prioritizes selecting samples with good radar echo coverage, and there are no duplicate frames between the training set and the testing set. The test results are shown in Table 4.

The experimental results show that PointPillars can extract effective spatial features from 4D imaging radar point clouds and complete vehicle 3D localization, verifying the feasibility of the constructed dataset for 3D object detection research based on 4D imaging radar.

## 6. Main Contributions and Conclusions

### 6.1. Main Contributions

(1) This article constructs a multimodal data-acquisition system and a three-dimensional traffic scene system based on the performance characteristics of 4D imaging radar. It establishes a complete data production process that includes data collection, synchronization, calibration, and semi-automatic 3D annotation. It also constructs a multimodal dataset covering eight typical scenes, containing 11,400 frames of data and 131,642 dynamic target annotations; effectively supplements the shortcomings of existing datasets in scene diversity and annotation scale; and provides a data foundation for the research of new perception algorithms for 4D imaging radar.

(2) To address the sparse point cloud problem in 4D imaging radar, a point cloud feature enhancement method is adopted. By improving the neighborhood construction method and proposing a distance-aware dynamic neighborhood strategy, the problem of distortion in feature extraction of distant targets and loss of local geometric information caused by fixed K-nearest neighbor schemes has been improved, enhancing the local expression ability of point clouds.

(3) In the process of 3D annotation, an adaptive iteration termination strategy is introduced to identify and optimize stagnant states based on changes in confidence, achieving early exit. At the same time, a maximum iteration upper limit and an optimal box fallback fault-tolerant mechanism are set to effectively improve the problems of boundary box mismatch and timing jitter, balancing annotation accuracy and computational efficiency.

### 6.2. Conclusions

This paper provides a detailed introduction to the construction technology of a multimodal 4D imaging radar dataset for three-dimensional traffic scenes. The framework encompasses the data-collection vehicle configuration, multi-sensor coordination mechanisms, hardware and software platform setup, design of typical traffic scenarios, point cloud data processing and validation, and multimodal fusion with large-model-based 3D annotation, forming a complete technical pipeline. Data collection, processing, and annotation have been completed for eight scenario categories, including overpasses, enclosed tunnels, complex intersections, and highways. This effort has resulted in the creation of a high-quality, multimodal, three-dimensional traffic scenario dataset.

The 4D imaging radar traffic scenario library established in this study can provide crucial data support for both academia and industry. It will facilitate research into novel perception algorithms tailored to 4D imaging radar’s characteristics, such as sparse point cloud completion and multi-object tracking and detection. Furthermore, it will promote the advancement of multimodal deep-fusion perception technologies involving high-resolution cameras, LiDAR, and 4D imaging radar, thereby laying a data foundation to enhance the safety of autonomous driving systems in complex urban environments.

Our follow-up study envisages expanding the scale and diversity of the dataset, enriching dynamic traffic data captured under adverse weather conditions, such as rain, snow, and fog, as well as under extreme lighting conditions, such as nighttime and strong backlighting. Efforts will also be directed toward optimizing 4D imaging radar perception models, fully exploiting their information advantages in velocity sensing and micro-Doppler feature extraction, thereby providing a robust benchmark and driving technological advancements in the development and validation of all-weather, high-reliability autonomous driving perception systems.

## Figures and Tables

**Table 1:** Comprehensive Comparison of Mainstream Radar Datasets for Autonomous Driving.

Dataset	Radar Type	Other Modalities	4D Point Cloud	Object Detection	Object Tracking	3D Annotations	Frames	Scenario Diversity	Weather Conditions
nuScenes	Low Res	Camera, LiDAR	×	√	√	√	40,000	Medium	Sunny/Rain/Night
RADIATE	Scanning	Camera, LiDAR	×	√	√	×	40,000	High	Sunny/Rain/Fog/Snow
RadarScenes	Low Res	Camera	×	√	√	×	121,287	Low	Sunny
RADIal	4D Imaging	Camera, LiDAR	√	√	×	×	8252	Low	Sunny
VoD	4D Imaging	Camera, LiDAR	√	√	√	√	8646	Low	Sunny/Rain/Dusk
TJ4DRadSet	4D Imaging	Camera, LiDAR	√	√	√	√	7757	Medium	Sunny/Night
Ours	4D Imaging	HD Camera, LiDAR	√	√	√	√	11,400	High	Sunny/Rain/Night

**Table 2:** Performance parameters and functions of 4D imaging radar.

Parameter	Range	Functionality
Maximum detection range	300–450 m	Long-range target detection and early warning
Range resolution	0.03–0.2 m	Adjacent target discrimination and clutter suppression
Velocity resolution	0.03–0.1 m/s	High-precision relative velocity measurement and moving target classification
Horizontal field of view	±75–±120°	Wide lateral coverage, enabling blind spot/lane change assistance
Vertical field of view	±5–±30°	Height information acquisition, identifying height limits/low-lying obstacles
Azimuth angle resolution	0.5–2°	High-precision horizontal angular resolution, target separation, and contour imaging
Elevation angle resolution	0.8–3°	Vertical dimension imaging, enabling 4D point cloud height perception

**Table 3:** Scenario design for evaluating 4D imaging radar point cloud data-acquisition performance.

Test Scenario	Number of Recorded Frames	Scene Characteristics	Core Validation Capabilities	Representative Scene Image
Urban complex intersection	1200	Dense moving/static heterogeneous targets	Resolution, classification, and tracking capabilities under high target density	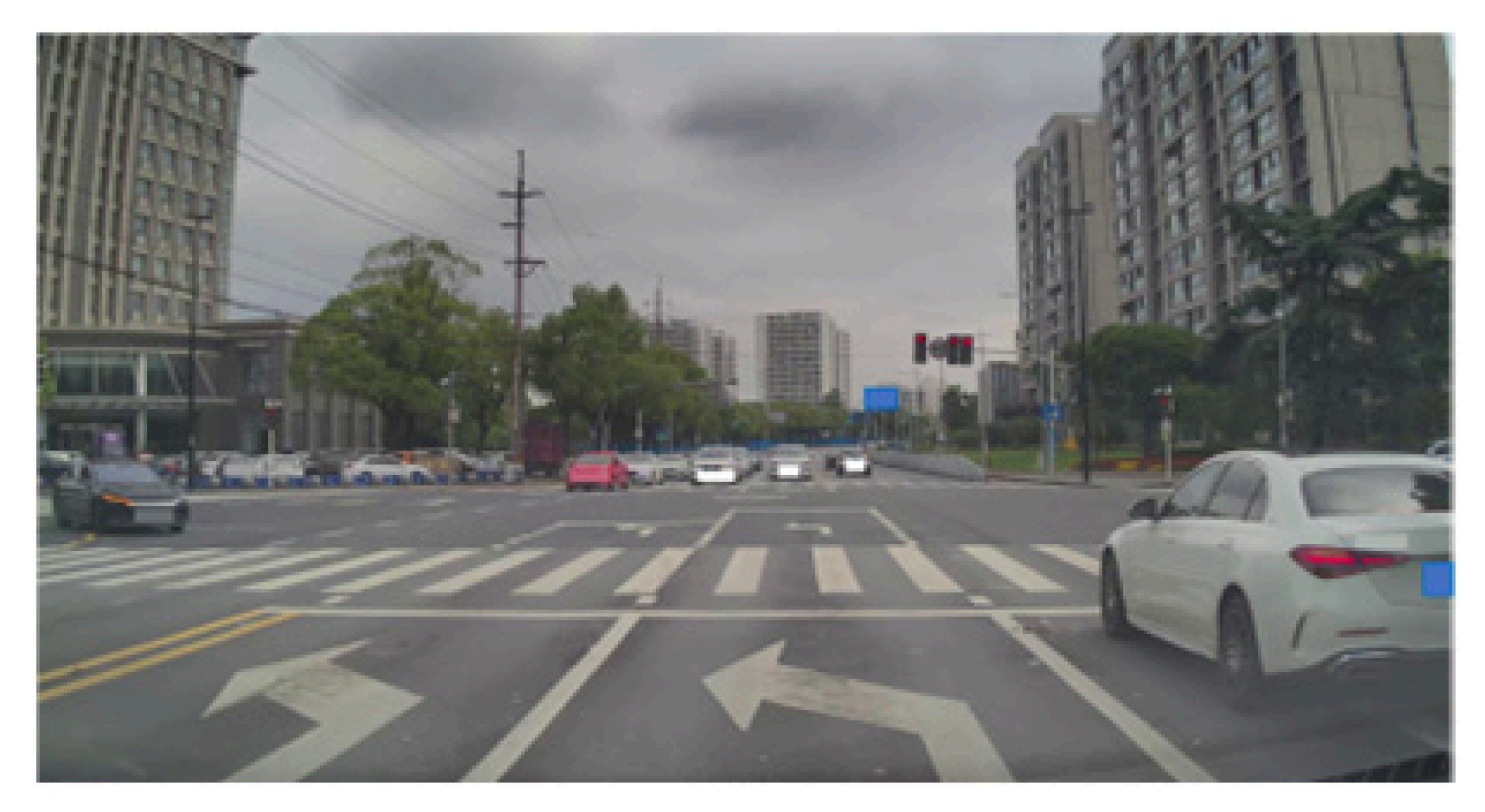
Multi-level overpass	1200	Multi-layer elevated structure with significant height differences	Accurate identification capability of target elevation information	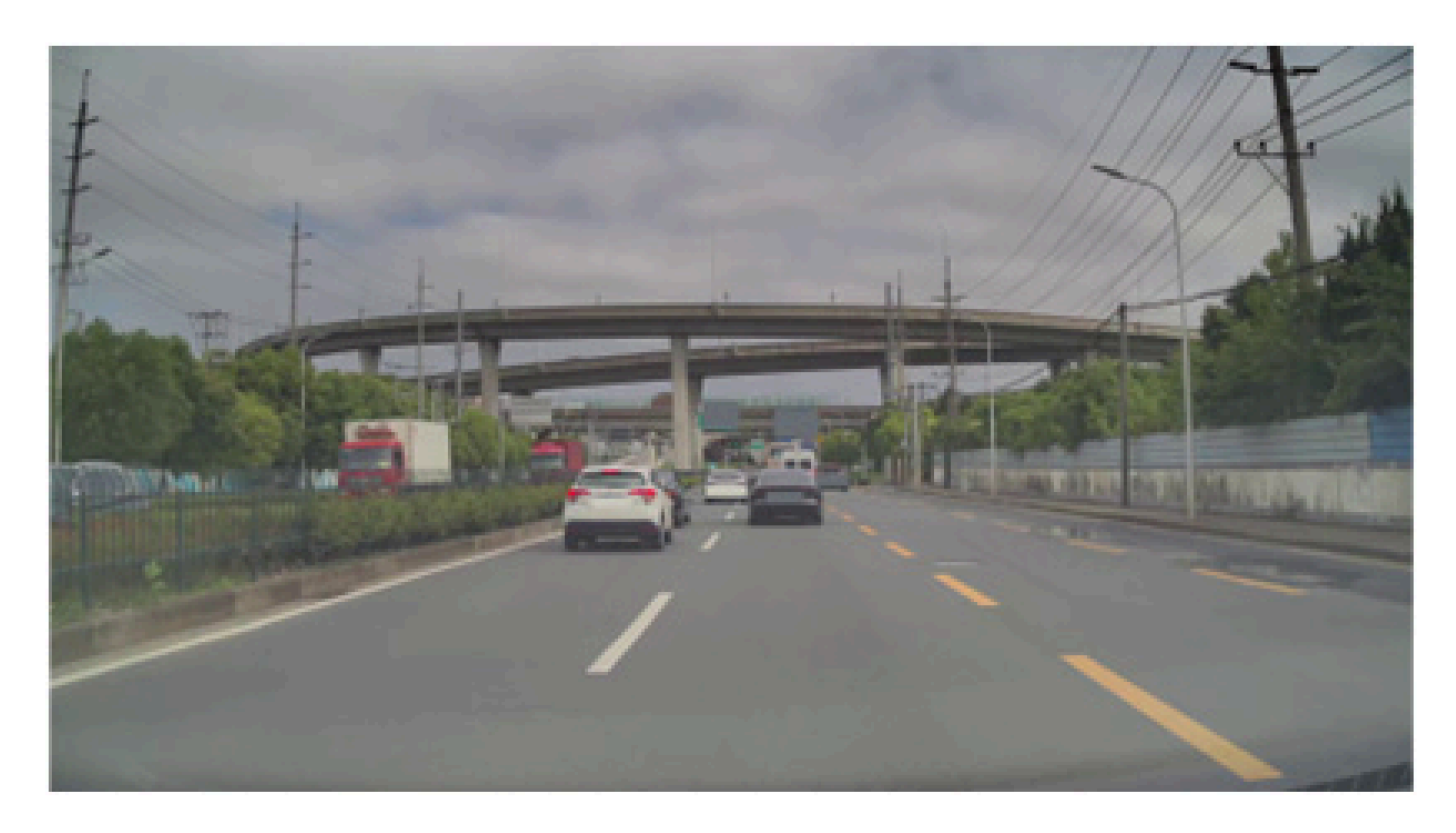
Enclosed tunnel	1200	Confined physical space with strong specular reflections	Signal processing and false alarm suppression performance in strong multipath environments	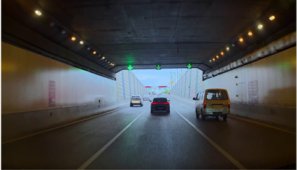
Undivided two-way lane	1200	Oncoming vehicles and their complex maneuvering states	Target trajectory and speed identification/tracking capability for oncoming vehicles	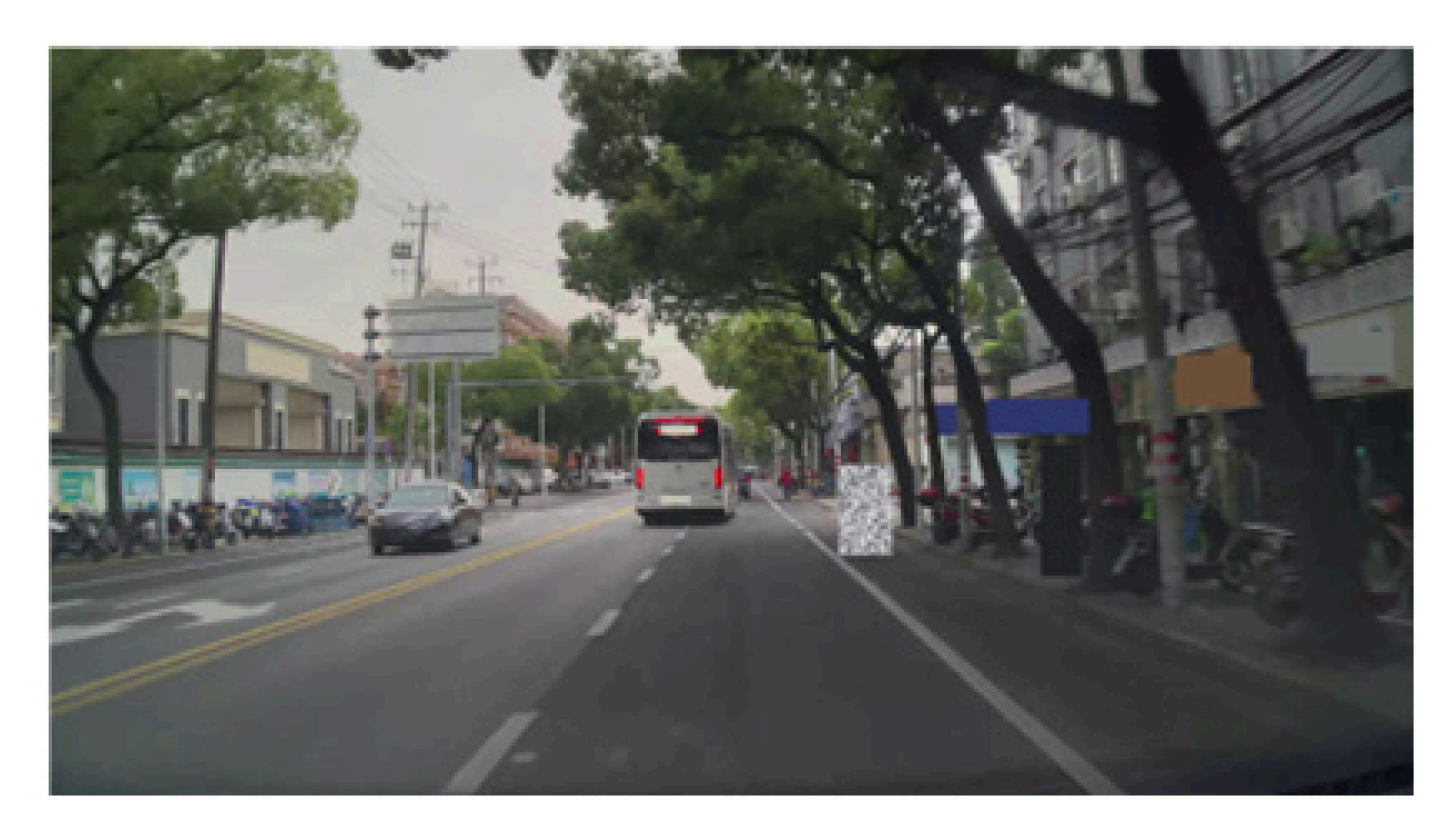
Highway	1200	Homogeneous and heterogeneous high-speed targets in a controlled-access setting	Trajectory tracking capability for distant targets with high Doppler shift	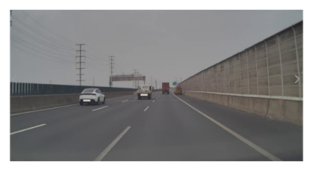
Highway weaving zone	1200	High-speed lane merging/cutting-in with multi-vehicle interactions	Trajectory and state identification of heterogeneous targets in complex interactive scenarios	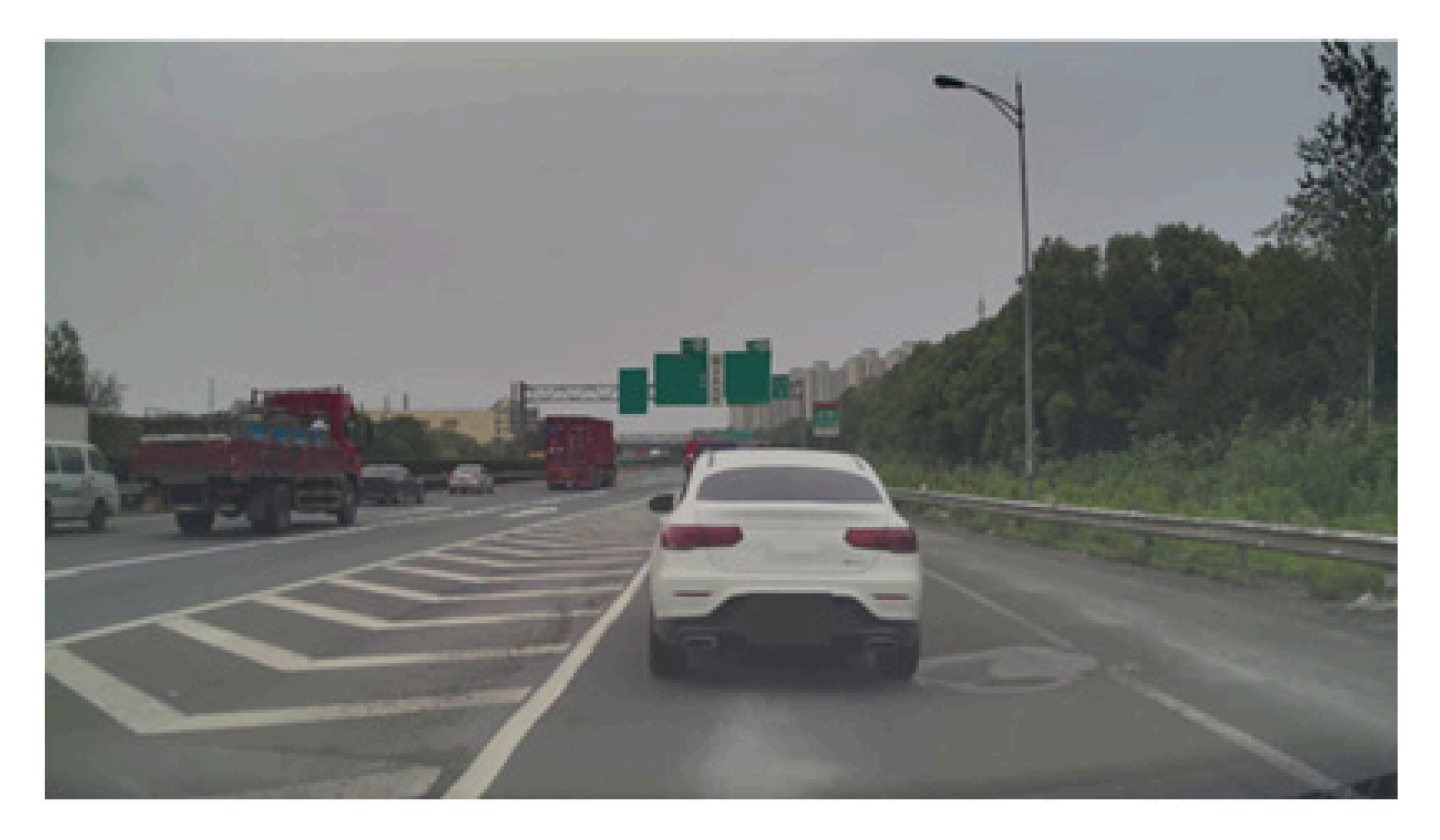
Congested toll plaza queue	1200	Coexistence and identification of slow-moving/stationary targets	Detection capability for targets with minor movements under a low signal-to-noise ratio	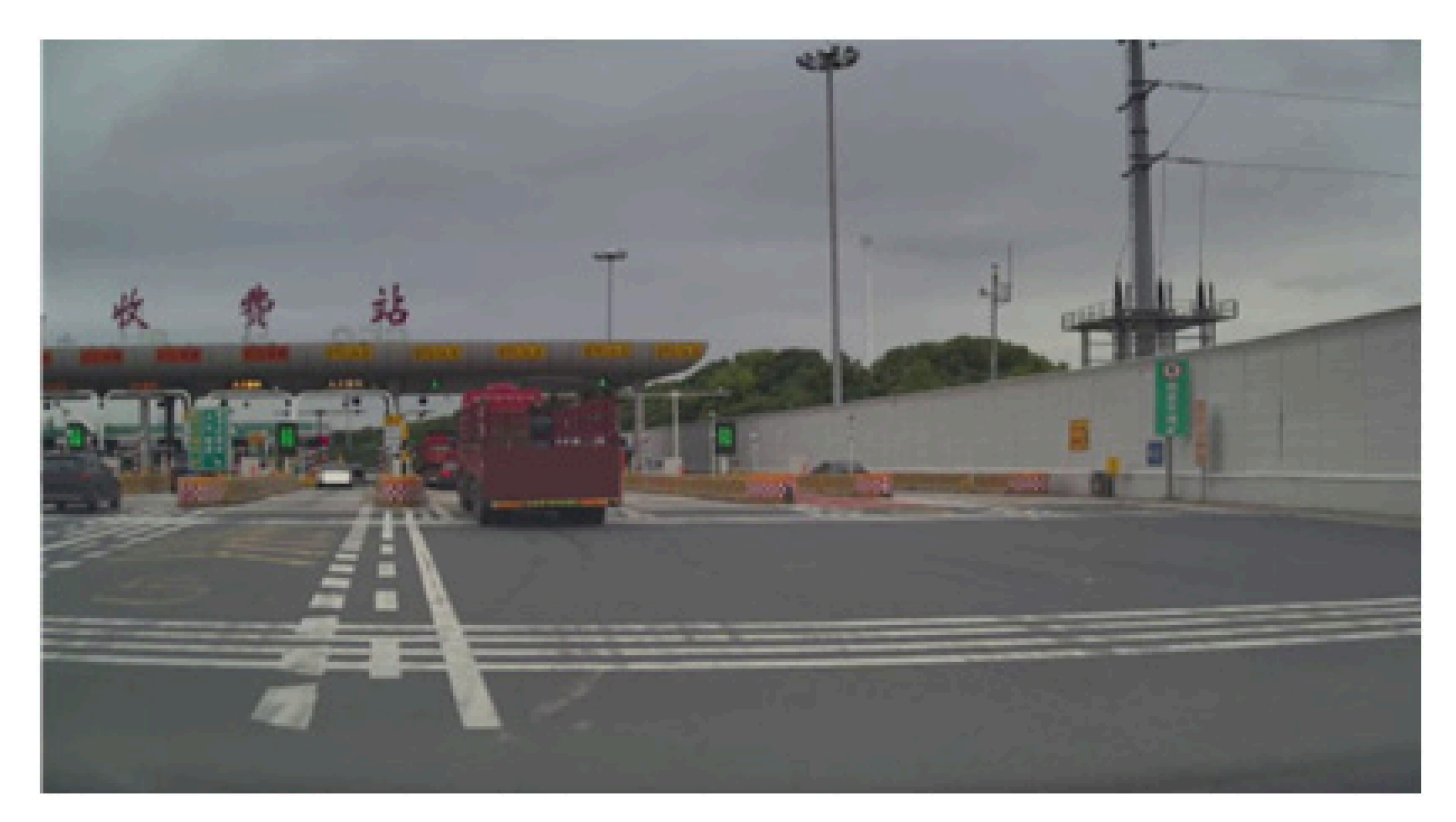
Enclosed vehicle proving ground	3000	Reproducible edge/corner cases	System robustness under critical events such as sudden maneuvers and occlusions	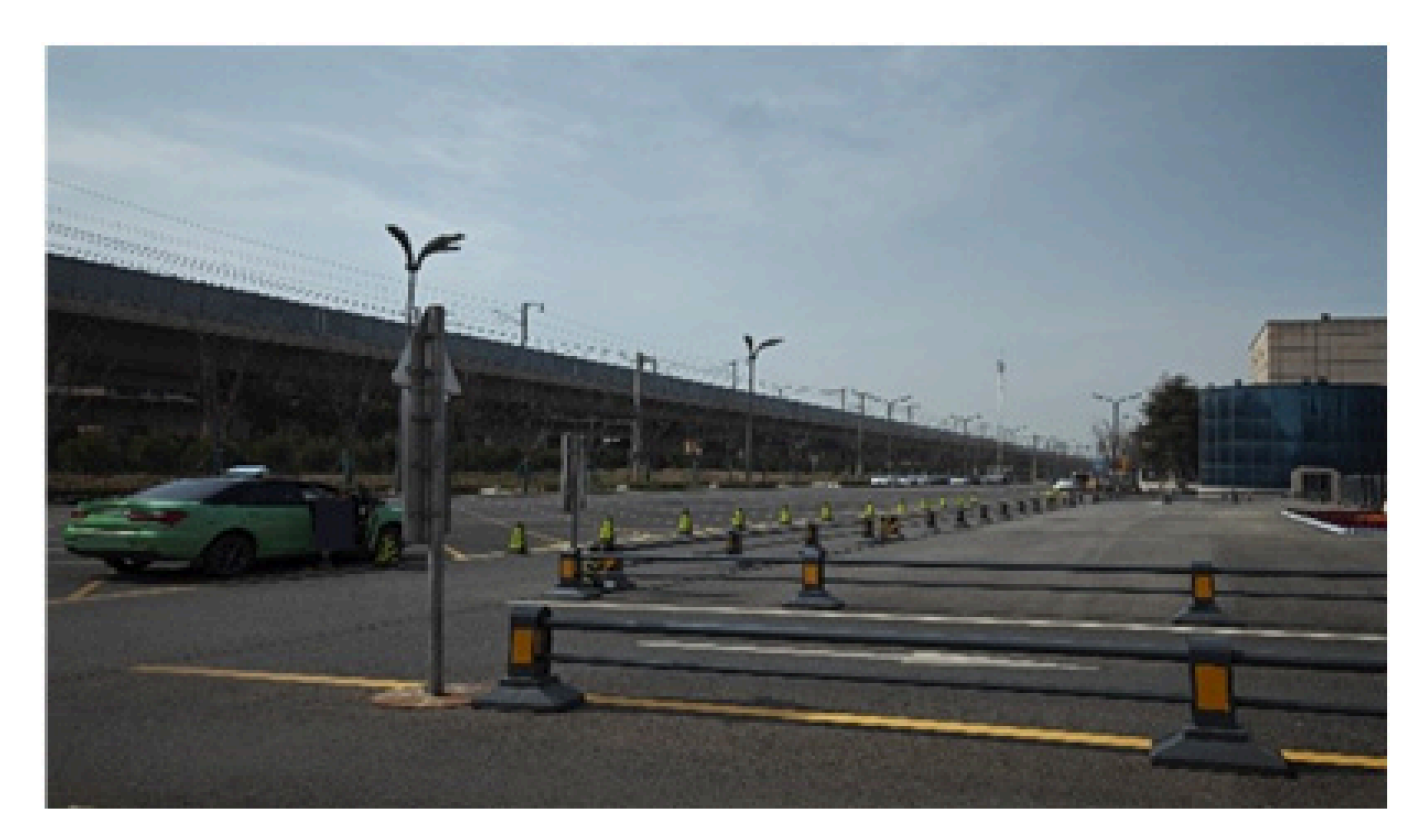

**Table 4:** Baseline experiment results.

Class	BEV AP@0.25	3D AP@0.25
Car	32.70	30.21
Truck	44.34	41.36
Mean	38.52	35.78

## Data Availability

The data supporting this study are available from the corresponding author upon request.
